# Garlic extracts prevent oxidative stress, hypertrophy and apoptosis in cardiomyocytes: a role for nitric oxide and hydrogen sulfide

**DOI:** 10.1186/1472-6882-12-140

**Published:** 2012-08-29

**Authors:** Xavier Lieben Louis, Ryan Murphy, Sijo Joseph Thandapilly, Liping Yu, Thomas Netticadan

**Affiliations:** 1 Canadian Centre for Agri-Food Research in Health and Medicine, Winnipeg,, R2H2A6, Canada; 2 Department of Physiology, University of Manitoba, Winnipeg,, R3E 0J9, Canada; 3 Agriculture and Agri-Food Canada, Winnipeg, R3T 2M9, Canada; 4 Department of Food Science, University of Manitoba, Winnipeg, R3T 2N2, Canada; 5 Heart Failure Research Laboratory, Canadian Centre for Agri-Food Research in Health and Medicine, R2020, St. Boniface Hospital Research Centre, 351 Tache Avenue, Winnipeg, Manitoba, R2H 2A6, Canada

## Abstract

**Background:**

In ancient times, plants were recognized for their medicinal properties. Later, the arrival of synthetic drugs pushed it to the backstage. However, from being merely used for food, plants are now been widely explored for their therapeutic value. The current study explores the potential of skin and flesh extracts from a hard-necked Rocambole variety of purple garlic in preventing cardiomyocyte hypertrophy and cell death.

**Methods:**

Norepinephrine (NE) was used to induce hypertrophy in adult rat cardiomyocytes pretreated with garlic skin and flesh extracts. Cell death was measured as ratio of rod to round shaped cardiomyocytes. Fluorescent probes were used to measure apoptosis and oxidative stress in cardiomyocytes treated with and without extracts and NE. Pharmacological blockade of nitric oxide (NO) and hydrogen sulfide (H_2_S) were used to elucidate the mechanism of action of garlic extracts. Garlic extract samples were also tested for alliin and allicin concentrations.

**Results:**

Exposure of cardiomyocytes to NE induced an increase in cell size and cell death; this increase was significantly prevented upon treatment with garlic skin and flesh extracts. Norepinephrine increased apoptosis and oxidative stress in cardiomyocytes which was prevented upon pretreatment with skin and flesh extracts; NO, and H_2_S blockers significantly inhibited this beneficial effect. Allicin and alliin concentration were significantly higher in garlic flesh extract when compared to the skin extract.

**Conclusion:**

These results suggest that both skin and flesh garlic extracts are effective in preventing NE induced cardiomyocyte hypertrophy and cell death. Reduction in oxidative stress may also play an important role in the anti-hypertrophic and anti-apoptotic properties of garlic extracts. These beneficial effects may in part be mediated by NO and H_2_S.

## Background

Cardiovascular disease and its associated mortality is responsible for an estimated 30% of all deaths worldwide [1]. Diseases such as hypertension, ischemic heart disease, cardiomyopathy and valvular heart disease which lead to the development of heart failure, stress the heart in the form of pressure or volume overload or a combination of both. The heart responds to this stress by increasing in size, a phenomenon termed as cardiac hypertrophy [2]. While this compensatory mechanism helps to accommodate the increased load on the heart, it eventually impairs cardiac function resulting in heart failure [3]. Current treatments for heart failure aimed at preventing the development of pathological hypertrophy have been moderately successful however, mortality due to heart failure is still on the rise [4]. Accordingly, the situation demands the need to explore complimentary or alternative therapies for heart failure. Whole foods or food derived compounds (from plants or animals) appear to be one such avenue.

The manner in which plants were valued is reverting back to its earlier status [5]. In ancient times, plants were recognized for their medicinal value, however, pushed to the backstage upon the arrival of synthetic drugs. From being merely used for food, plants have been widely explored for their medicinal values. With the science of plant components being revealed, plants are returning into the mainstream of therapeutics. Garlic (*Allium sativum*) is one such plant which is believed to have originated around 6000 years ago, and was held in high regard as a therapeutic agent against cardiovascular pathologies [6]. It was also known to have antimicrobial properties and offer protection against cancer and other health risks. In the earlier days garlic was also used as a flavoring agent in food.

Earlier studies with garlic were mainly aimed at the therapeutic potential of whole garlic homogenates [7]. However, there is no information on the potential of garlic skin; a papery covering surrounding the individual cloves and a by-product of garlic processing. There are only two reports on the effectiveness of garlic in preventing the development of cardiac hypertrophy in animal models of hypertension and diabetes [8,9] however, the mechanisms underlying garlic effects are not explored. Lastly, no study has examined the potential of a variety of hard necked Rocambole purple garlic developed in Manitoba, Canada. In view of the above mentioned gaps in the literature, the current study aims to determine the anti-hypertrophic potential of extracts from Manitoban purple garlic flesh and skin, and explore the underlying mechanisms of action. For this purpose, the effects of garlic extracts were studied on an established cell model of pathological hypertrophy. We also aim to study whether nitric oxide (NO) and hydrogen sulfide (H_2_S) has a role in mediating the medicinal properties of purple garlic extracts.

## Methods

All experimental protocols used in this study were approved by the University of Manitoba Animal Care Committee and are in agreement with the Canadian Council on Animal Care and Use of Experimental Animals (vol. 1, 2nd ed., 1993).

All chemicals used in this study were purchased from Sigma-Aldrich, Ontario, Canada.

### Cardiomyocyte isolation

Ventricular myocytes were isolated from 12-week old male Sprague- Dawley rats (200-250 g) as described previously [10]. In brief, an intramuscular injection of a mixture of ketamine (90 mg/kg) and xylazine (10 mg/kg) was used to anesthetize the animal. Excised hearts were quickly transferred to a langendorf perfusion apparatus and perfused with calcium (Ca^2+^) free buffer containing (in mM); 90 NaCl, 10 KCl, 1.2 KH_2_PO_4_, 5 MgSO_4_.7H_2_O, 15 NaHCO_3_, 30 taurine and 20 glucose for 5 min. The perfusion medium was then switched to Ca^2+^ free buffer containing collagenase (0.05%) and bovine serum albumin (0.2%). After 30 min ventricles were cut into small pieces, incubated in a 37°C waterbath and separated into individual cardiomyocytes by slow pipetting. Cardiomyocytes were then suspended in buffer containing Ca^2+^ and the cells were allowed to settle. The supernatant was then replaced with Ca^2+^ buffers containing higher concentration of calcium (150 mM). This step was repeated twice to increase the extracellular Ca^2+^ concentration to 500 mM and then to 1.2 M. Cells were finally resuspended in medium-199 (M199) containing 10% fetal bovine serum supplemented with 5 mM taurine, 2 mM carnitine, 1 mM creatine, 1 mM insulin and, and transferred to laminin coated culture dishes. After 2 hours of incubation in a CO_2_ incubator (5% CO_2_ and 95% O_2_), the existing medium was replaced with serum free 1X (pH 7.4) M199 (Invitrogen, ON, Canada) supplemented with 5 mM taurine, 2 mM carnitine, 1 mM creatine and 1 mM insulin. All cells were incubated for 24 hours at 37°C before starting any experimental procedure. The medium was not refilled for any experiment employed in this study.

### Garlic extracts preparation

Five grams of garlic flesh was ground and mixed with 60 mL of distilled water for 30 minutes. The mixture was filtered using coarse filter paper to produce the extracts used in the study. Garlic skin extract was also prepared following the same method.

### Analyses of allicin and alliin concentrations

Garlic extract samples were prepared as described above. High performance liquid chromatography (HPLC) analyses of allicin and alliin in garlic flesh and skin extract samples were performed by Silliker, JR laboratories, Burnaby, BC, Canada.

### Effect of garlic extracts on norepinephrine (NE) induced cardiomyocyte hypertrophy

Stock solutions of NE (1 mM) were prepared in 15 mM ascorbic acid. In this experiment, cardiomyocytes were pre-treated with different volumes of garlic extracts (4, 10 and 20 μl per 4 mL of cell culture medium) for 30 min and then co-incubated with NE (0.25 μM) for 24 hours. Phase contrast images taken by an Olympus microscope (Olympus Canada Inc. ON, Canada) were used to measure surface area of individual cardiomyocyte using ImageJ software. A total of 100 cells from 3 different animals were analyzed for determining the morphological changes induced by NE. The most effective concentration was used for further experiments.

### Effect of NO and H_2_S inhibitors on garlic extract mediated anti-hypertrophic action on cardiomyocytes

Pharmacological blockers of NO, NG-nitro-L-arginine methyl ester (LNAME) and H_2_S, propargylglycine (PAG) were used to determine the role of these gaseous molecules in the beneficial action of garlic extracts. Stock solution of LNAME (100 mM) and PAG (300 mM) were prepared in double distilled water. Cells were treated with LNAME and PAG prior to the addition of extracts and NE. Briefly, 24 hours post isolation, cardiomyocytes were treated with 100 μM of LNAME and 200 μM of PAG for 1 hour. Garlic extracts (20μL per 4 mL M199) were then added to the medium and incubated for 30 min. The cells were then stimulated with NE (0.25 μM). After 24 hours of incubation, cardiomyocyte surface area was measured as described earlier.

### Cell death and apoptosis measurements

Cardiomyocytes were incubated with different volumes of garlic extracts (4, 10 and 20 μl per 4 mL of cell culture medium) for 30 minutes and further exposed to NE as described earlier. To block NO and H_2_S, cardiomyocytes were pretreated with corresponding pharmacological blockers as described in the previous experiment. Round shaped cardiomyocytes were considered dead and cell death was analyzed by calculating the ratio of round to rod shaped cells. After 24 hours, phase contrast images were captured using Olympus microscope. A total of 200 cells from three independent cardiomyocyte isolations were used for the analysis using ImageJ software. Apoptosis was estimated using hoechst pentahydrate (bis-benzimide) stain (Invitrogen, ON, Canada). Cardiomyocytes were pretreated with 20 μl (an optimal dose obtained from the experiment mentioned above) of garlic skin and flesh extracts and then exposed to NE. Some cardiomyocytes were also pretreated with pharmacological blockers for NO and H_2_S. After 24 hours of NE exposure cells were washed with warm 1X phosphate-buffered saline (PBS) and added 2 μM hoechst stain. Cells were incubated with hoechst stain for 10 min in the dark and further cells were imaged using Olympus fluorescent microscope. Cells with condensed nuclei were counted to measure apoptosis.

### Oxidative stress measurement

Cardiomyocytes treated with different volumes of garlic flesh/skin extracts (4, 10 and 20 μl per 4 mL of cell culture medium) and further incubated with NE for 24 hours. Another experiment was conducted in which some cardiomyocytes were exposed to LNAME and PAG for 1 hour prior to addition of 20 μl (an optimal dose obtained from the experiment mentioned above) of the extracts. Post incubation, cells were washed with warm PBS and incubated for 30 minutes with 5 μM solution of 5-(and-6)-chloromethyl-2',7'-dichlorodihydrofluorescein diacetate, acetyl ester (Invitrogen Corp. CA, USA); a fluorescent indicator for intracellular detection of reactive oxygen species. After incubation, fluorescent images were captured using Olympus fluorescent microscope. Fluorescence intensity of captured images was measured using Image-pro analytical software (Media cybernetics, U.S.A).

### Statistical analysis

Data were expressed as mean + standard error (S.E.M). Statistical analysis of data was performed by applying one-way analysis of variance (ANOVA) followed by tukey post-hoc test. P value <0.05 was considered statistically significant.

## Results

### Analyses of allicin and alliin concentrations

Garlic flesh extract had very high levels of allicin (0.233 mg/mL) and alliin (0.04 mg/mL) in comparison to the levels detected in garlic skin extract [allicin (<0.002 mg/mL) and alliin (0.004 mg/mL)] (Table 1).

**Table 1:** Data showing the concentration of allicin and alliin in garlic flesh and skin extracts

**Sample ID**	**Allicin (mg/mL)**	**Alliin (mg/mL)**
Garlic flesh	0.233	0.04
Garlic skin	<0.002	0.004

### Effect of garlic skin and flesh extract on NE induced increase in cardiomyocyte size

**Figure 1:**
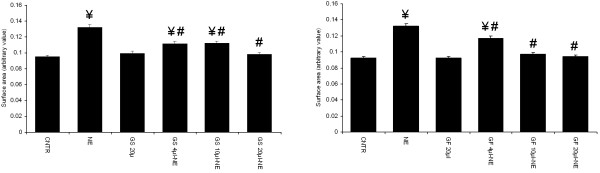
**Dose dependent anti-hypertrophic effect of garlic extracts. a.** garlic skin (GS) and **b.** garlic flesh (GF). Data are mean ± S.E.M. n = 100 cells from three independent experiments. CNTR, control; NE, norepinephrine.

Cardiomyocytes stimulated with 0.25 μM NE for 24 hours induced a significant increase in surface area of the cells, when compared to unstimulated control cardiomyocytes. Garlic skin and flesh extracts had dose dependent effect on NE induced increase in cardiomyocyte surface area (in comparison to untreated control cardiomyocytes stimulated with NE). Twenty microlitres of skin extract, per 4 mL of cell culture medium significantly prevented the increase in cell size. The most effective doses of flesh extract were 10 and 20 μl per 4 mL of cell culture medium. Garlic skin (20 μl) and flesh (20 μl) extracts alone did not have any effect on control cardiomyocytes (Figure 1a & b).

### Effect of LNAME and PAG on garlic extracts mediated anti-hypertrophic action on cardiomyocytes

**Figure 2:**
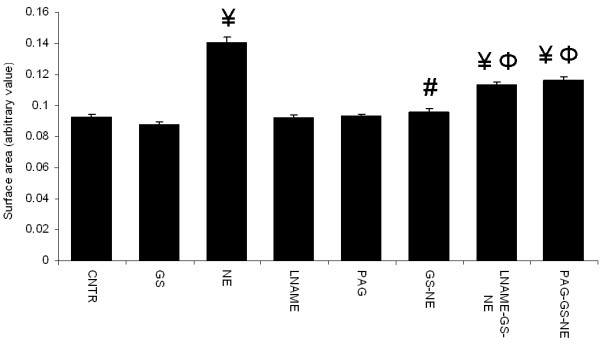
**Effect of pharmacological blockade of nitric oxide and hydrogen sulfide on antihypertrophic action of garlic skin (GS) extract.** Data are mean ± S.E.M. n = 100 cells from three independent experiments. CNTR, control; NE, norepinephrine; LNAME, NG-nitro-L-arginine methyl ester; PAG, propargylglycine.

**Figure 3:**
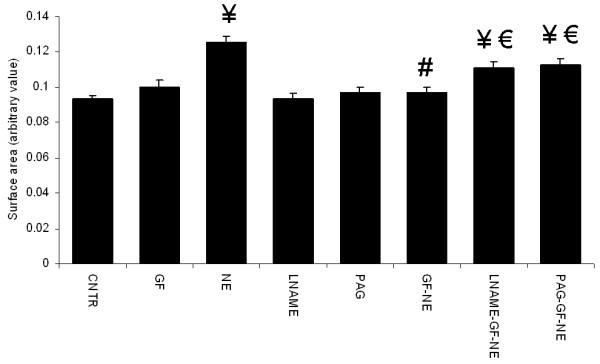
**Effect of pharmacological blockade of nitric oxide and hydrogen sulfide on antihypertrophic action of garlic flesh (GF) extract.** Data are mean ± S.E.M. n = 100 cells from three independent experiments. CNTR, control; NE, norepinephrine; LNAME, NG-nitro-L-arginine methyl ester; PAG, propargylglycine.

Cardiomyocytes stimulated with 0.25 μmol NE for 24 hours induced a significant increase in surface area of the cells in comparison to unstimulated control cells; pretreatment with garlic extracts prevented the NE induced increase in cell size. Pre-incubation with LNAME and PAG significantly reduced the anti-hypertrophic effects of both garlic skin and flesh extracts on NE stimulated cardiomyocytes; LNAME or PAG by itself did not have any effect on control cardiomyocytes (Figures 2 &3).

### Cell death and apoptosis measurements

**Figure 4:**
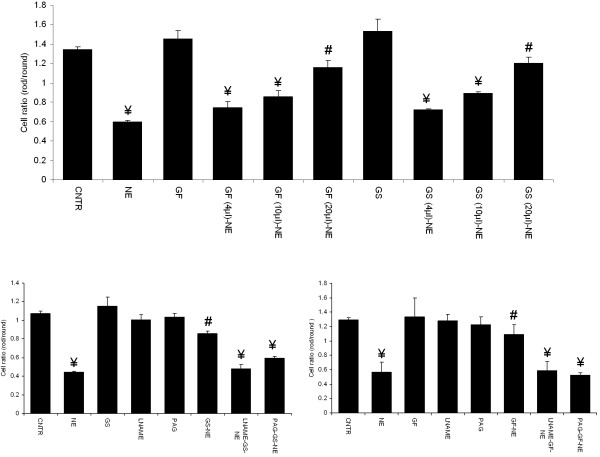
**Effect of garlic extracts on NE induced cell death. a.** garlic skin (GS), **b.** garlic flesh (GF). Data are mean ± S.E.M. n = 100 cells from three independent experiments. CNTR, control; NE, norepinephrine; LNAME, NG-nitro-L-arginine methyl ester; PAG, propargylglycine.

**Figure 5:**
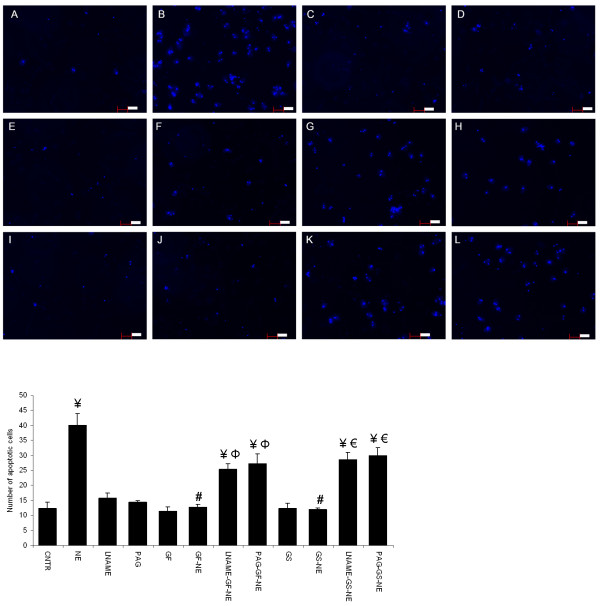
**Hoechst staining showing effect of garlic extracts on NE induced apoptosis. a.** Representative set of image from three independent experiments. **A.** Control, **B.** NE, **C.** LNAME, **D.** PAG, **E.** GF, **F.** GF-NE, **G.** LNAME-GF-NE, **H.** PAG-GF-NE, **I.** GS, **J.** GS-NE, **K.** LNAME-GS-NE, **L.** PAG-GS-NE. Staining technique has been optimized to show only cells with condensed nuclei. **b.** Graphical data showing the effect of garlic extracts cardiomyocyte apoptosis. Data are mean ± S.E.M. n = 3. Length of bar = 100 μm. CNTR, Control; NE, norepinephrine; LNAME, NG-nitro-L-arginine methyl ester; PAG, propargylglycine; GS, garlic skin extract; GF, garlic flesh extract.

Round to rod cell ratio was significantly decreased in cardiomyocytes exposed to NE when compared to control group. A dose dependent effect of garlic extracts were observed wherein, only the 20 μl dose of garlic skin and flesh extracts prevented this increase in cell death (Figure 4a). Treatment with LNAME and PAG abolished garlic extracts (20 μl per 4 ml culture medium) mediated prevention of NE induced cell death. Blockers, LNAME and PAG by itself did not have any effect on control cardiomyocytes (Figure 4b & c). Hoechst staining showed that exposure to NE increased number of cells with condensed nuclei when compared to control cardiomyocytes; treatment with garlic skin and flesh extracts significantly prevented this increase. Pretreatment with NO and H_2_S blockers blunted the beneficial effect of garlic extracts in preventing cardiomyocyte apoptosis (Figure 5a & b).

### Oxidative stress measurement

**Figure 6:**
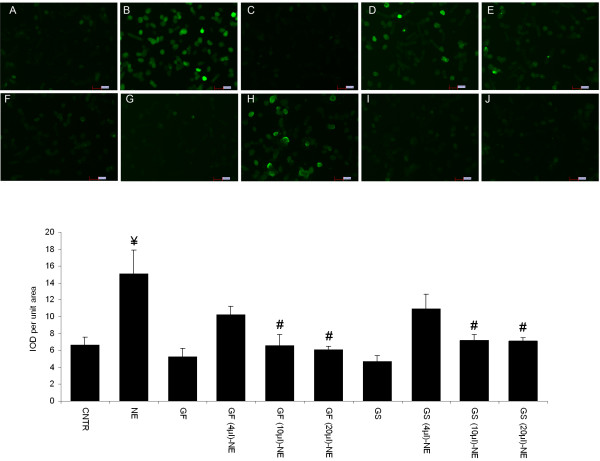
**Measurement of oxidative stress. a.** Representative set of image from three independent experiments. **A.** Control, **B.** NE, **C.** GF, **D.** GF(4 μl)-NE, **E.** GF(10 μl)-NE, **F.** GF(20 μl)-NE, **G.** GS, **H.** GS(4 μl)-NE, **I. **GS(10 μl)-NE, **J.** GS(20 μl)-NE. **b.** Fluorescent intensity data showing the effect of garlic extracts on oxidative stress in cardiomyocytes. Data are mean ± S.E.M. n = 3. Length of bar = 100 μm. IOD, image optical density; CNTR, Control; NE, norepinephrine; GS, garlic skin extract; GF, garlic flesh extract.

**Figure 7:**
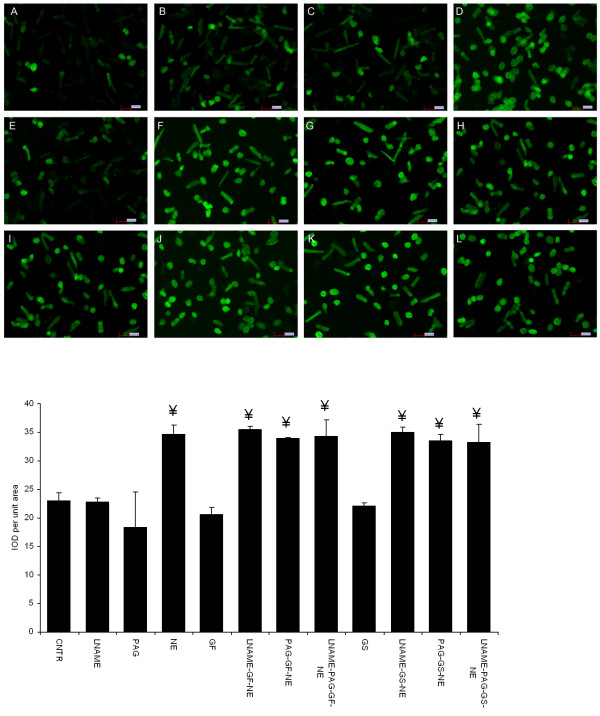
**Effect of LNAME and PAG on garlic extracts mediated reduction in oxidative stress a.** Representative set of image from three independent experiments. **A.** CNTR, **B.** LNAME, **C.** PAG, **D.** NE, **E.** GF, **F.** LNAME-GF-NE, **G.** PAG-GF-NE, **H.** LNAME-PAG-GF-NE, **I.** GS, **J.** LNAME-GS-NE, **K.** PAG-GS-NE, **L.** LNAME-PAG-GS-NE. **b.** Fluorescent intensity data showing the effect of pharmacological blockers on garlic mediated reduction in oxidative stress. Data are mean ± S.E.M. n = 3. Length of bar = 100 μm. IOD, image optical density; CNTR, Control; NE, norepinephrine; LNAME, NG-nitro-L-arginine methyl ester; PAG, propargylglycine; GS, garlic skin extract; GF, garlic flesh extract.

Oxidative stress, measured as fluorescence intensity was significantly increased in cardiomyocytes exposed to NE, when compared to the control. Treatment with 10 and 20 μl garlic flesh and skin extracts prevented this increase in NE induced oxidative stress. (Figure 6a & b). However, pretreatment with LNAME and PAG prevented the garlic skin and flesh extracts (20 μl per 4 ml culture medium) mediated reduction in oxidative stress. Simultaneous blockade of NO and H_2_S did not show any added effect when compared to the independent exposure to LNAME and H_2_S. Pretreatment with LNAME and PAG alone did not have any effect on normal cardiomyocytes (Figure 7a & b).

## Discussion

Intake of garlic has been reported earlier to reduce blood pressure and improve cardiovascular abnormalities, and many studies thereafter explored the cardioprotective properties of garlic [11,12]. These new findings resulted in an increase in demand for garlic products in the market and encouraged the development of wide varieties of garlic with better quality and which would be grown more efficiently. In this study, we used extracts from a variety of hard necked Rocambole purple garlic developed locally in Manitoba, Canada. This new garlic variety is better adapted to harsh climate and has superior disease resistance qualities. In general, the purple garlic has a colored skin which possibly indicates the presence of a different set of bioactive compounds when compared to the normal variety of garlic with white skin. Furthermore, no study has explored the medicinal properties of garlic skin in any form. Accordingly, in this study we used skin and flesh extracts from a purple variety of garlic to examine their potential in preventing cardiomyocyte hypertrophy and cell death. Neonatal and adult cardiomyocytes are the two major cell culture models used to study heart diseases. However, neonatal cardiomyocytes are not terminally differentiated and differ in hypertrophic signaling in comparison to adult cardiomyocytes [13]. Therefore, we used adult rat cardiomyocytes as it is the most appropriate experimental *in vitro* model to study heart diseases which are prevalent in adult stages of life.

Norepinephrine is an important neurohormone. It acts on the heart by activating cellular signaling promoting increased heart rate and enlargement of cardiomyocytes [14]. However, chronic exposure to elevated levels of NE results in cardiac damage, and high levels of NE in blood is considered a pathological marker for heart failure [15]. We have recently reported [10] the use of this model to study cardiomyocyte hypertrophy that mimics pathological remodeling due to heart disease in humans. In this study, we report that both garlic skin and flesh extracts are equally beneficial in preventing NE induced cardiomyocyte hypertrophy.

Garlic is known to increase cellular levels of H_2_S, an indigenous gaseous molecule that is reported to prevent progression of cardiac hypertrophy to heart failure [16]. Garlic also stimulates production of NO, another gaseous molecule that is widely accepted for its role in protecting the tissues including heart from pathologic damage [17]. NO and H_2_S has been reported to improve survival after incidence of cardiac damage [18]. These two gaseous molecules, as cellular messengers also play a critical role in modulating cardiomyocyte contractile functions [19]. Furthermore, H_2_S and NO cross talk has been reported to protect cells and tissues [20]. Accordingly, we hypothesized that the beneficial effects of garlic extracts on cardiomyocyte hypertrophy could in part be mediated by these two molecules. Consistent, with this view, we found that pretreatment of cardiomyocytes with pharmacological blockers for H_2_S and NO significantly, blocked the anti-hypertrophic action of both garlic skin and flesh extracts. Based on our result we speculate that, upregulation of H_2_S and NO may be mediating some of the mechanisms underlying the antihypertrophic action of garlic observed in earlier studies conducted on animal models of cardiac hypertrophy induced by hypertension [9] and diabetes [8].

Cardiac hypertrophy can be reversed in certain conditions of heart disease [21]. However, prolonged cardiac hypertrophy will lead to a decompensatory stage where the heart function deteriorates and progress towards heart failure [3]. Increased cell death has been observed as a characteristic of this stage [22]. Cell death accelerates the spread of heart tissue damage from one region of the heart to other areas. In order to prevent this progression, it is important to prevent cell death, and the development of heart failure [23]. In this regard, NE has been reported to play a major role in the process of promoting cell death [24]. Data from this study show that both garlic skin and flesh extracts prevented NE induced cell death and may therefore be beneficial in preventing cell death and progression of cardiac hypertrophy into heart failure. The cellular mechanism underlying these positive results with garlic extracts was next examined. Similar to the results observed on cardiomyocyte hypertrophy, beneficial effect of both skin and flesh extracts on cell death were blunted, when cardiomyocytes were exposed to NO and H_2_S blockers.

It is well documented that oxidative stress is the genesis of both cardiac hypertrophy and cell death [25]. We have earlier reported that oxidative stress is increased in animal models of hypertension induced cardiac hypertrophy and impaired cardiac function [26,27]. Also, NE is known to induce oxidative stress in rats [28]. Earlier studies have showed that reduction in oxidative stress as an important cellular mechanism by which many plant based products exert their beneficial effects [29]. Similarly, garlic and its ingredient molecules are known for their antioxidant properties [30]. Examination of oxidative stress in our model showed that garlic skin and flesh extracts were equally efficient in preventing NE induced oxidative stress. Experiment with different concentrations of garlic skin and flesh extracts showed that irrespective of the ineffectiveness of the 10 μl dose against preventing hypertrophy, it prevented NE induced increase in oxidative stress. This suggests that mechanisms other than preventing increase in oxidative stress may be involved in the antihypertrophic action of garlic extracts. Accordingly, it is only reasonable to speculate that in this *in vitro* model, oxidative stress may have a role in mediating effects of garlic flesh extract against preventing cardiomyocyte hypertrophy and cell death. However, it is interesting to know what mediates this antioxidant property of garlic. As mentioned earlier, many important medicinal properties of garlic are mediated by NO and H_2_S. It is also reported that NO and H_2_S abates oxidative stress through different mechanisms [31]. Therefore, we tested the effect of NO and H_2_S blockade on garlic extracts induced reduction in oxidative stress. Our data showed that the blockade of NO and H_2_S prevented garlic extracts mediated reduction in oxidative stress. This suggests that these gaseous molecules could be directly or indirectly promoting the antioxidant properties of garlic.

In addition, garlic bioactives primarily allicin and alliin have been shown to possess cardioprotective properties [32-35]. Results from our analyses show that garlic flesh extract had very high amounts of allicin and alliin when compared with the garlic skin extract. The difference in allicin and alliin concentrations (between flesh and skin extracts) suggest that these bioactives may not be the major players contributing to the beneficial effects of garlic observed in this study. Also, it is possible that the two extracts may deliver cardiomyocyte protection through separate mechanisms.

## Conclusion

Skin and flesh extracts of purple variety of garlic is beneficial in preventing cardiomyocyte hypertrophy and cell death. The skin and flesh extracts were also beneficial in preventing oxidative stress, the genesis of above mentioned deleterious process. Our data also sheds some light into the cellular mechanism of action of garlic extracts as we report that the protective effects may be in part mediated by the endogenous gaseous molecules, H_2_S and NO. However, further studies are required to confirm these beneficial effects and the exact role NO and H_2_S in delivering medicinal properties of garlic. In addition, garlic extract analyses done in our study suggests that the major garlic bioactives, allicin and alliin, may or may not be involved in the observed cardiomyocyte protection. Another important finding of this study is that the garlic skin has medicinal properties, and is equally beneficial as the garlic flesh in preventing cardiomyocyte hypertrophy and cell death. Accordingly, the garlic skin, which is usually discarded in garlic processing, could be used in garlic products thus promoting traditional garlic growers and industry.

## Competing interests

Prairie Garlic Ltd. Manitoba, Canada, provided material, labor and transportation for the development of this new variety of garlic. However, the organization had no role in design or the implementation of this study, as well as, it had no financial involvement in this study. Further, the authors have no financial involvement with any organization or entity with a financial interest in the subject or materials discussed in the manuscript.

## Authors’ contributions

XLL and TN designed the research project. RM prepared garlic extracts and performed dose study of garlic extracts. XLL performed all other experiments mentioned in this manuscript. SJT assisted in morphological assessments and statistical analysis. LY performed cardiomyocyte isolation for all the experiments. XLL and TN prepared the manuscript. All authors read and approved the final manuscript.

## Pre-publication history

The pre-publication history for this paper can be accessed here:

http://www.biomedcentral.com/1472-6882/12/140/prepub
